# *N*-(2,4,6-Trichloro­phen­yl)maleamic acid

**DOI:** 10.1107/S1600536811029436

**Published:** 2011-07-30

**Authors:** K. Shakuntala, Sabine Foro, B. Thimme Gowda

**Affiliations:** a Department of Chemistry, Mangalore University, Mangalagangotri 574 199, Mangalore, India; b Institute of Materials Science, Darmstadt University of Technology, Petersenstrasse 23, D-64287 Darmstadt, Germany

## Abstract

In the crystal structure of the title compound, C_10_H_6_Cl_3_NO_3_, the conformation of the amide bond is *trans*. The C=O and O—H bonds of the acid group are in the relatively rare *anti* position to each other. This is a consequence of the intra­molecular O—H⋯O hydrogen bond donated to the amide carbonyl group stabilizing the mol­ecular structure. In the crystal, inter­molecular N—H⋯O hydrogen bonds link the mol­ecules into zigzag chains along the *c* axis.

## Related literature

For studies on the effects of substituents on the structures and other aspects of *N*-(ar­yl)-amides, see: Arjunan *et al.* (2004 ▶); Bhat & Gowda (2000 ▶); Gowda *et al.* (2000 ▶, 2009 ▶); Lo & Ng (2009 ▶); Prasad *et al.* (2002 ▶), and on *N*-(ar­yl)-methane­sulfonamides, see: Jayalakshmi & Gowda (2004 ▶). For modes of inter­linking carb­oxy­lic acids by hydrogen bonds, see: Leiserowitz (1976 ▶).
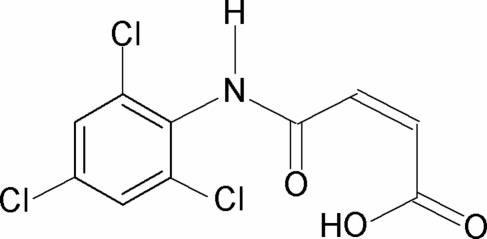


## Experimental

### 

#### Crystal data

C_10_H_6_Cl_3_NO_3_
*M* *_r_* = 294.51
Monoclinic, 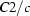

*a* = 21.928 (3) Å
*b* = 8.2678 (8) Å
*c* = 13.248 (2) Å
β = 99.08 (1)°
*V* = 2371.7 (5) Å^3^
*Z* = 8
Mo *K*α radiation
μ = 0.77 mm^−1^
*T* = 293 K
0.44 × 0.44 × 0.40 mm

#### Data collection

Oxford Diffraction Xcalibur diffractometer with a Sapphire CCD detector
Absorption correction: multi-scan (*CrysAlis RED*; Oxford Diffraction, 2009 ▶) *T*_min_ = 0.729, *T*_max_ = 0.749
4862 measured reflections
2436 independent reflections
2000 reflections with *I* > 2σ(*I*)
*R*_int_ = 0.012

#### Refinement

*R*[*F* ^2^ > 2σ(*F* ^2^)] = 0.035
*wR*(*F* ^2^) = 0.094
*S* = 1.08
2436 reflections
161 parameters
2 restraints
H atoms treated by a mixture of independent and constrained refinement
Δρ_max_ = 0.55 e Å^−3^
Δρ_min_ = −0.44 e Å^−3^

### 

Data collection: *CrysAlis CCD* (Oxford Diffraction, 2009 ▶); cell refinement: *CrysAlis RED* (Oxford Diffraction, 2009 ▶); data reduction: *CrysAlis RED*; program(s) used to solve structure: *SHELXS97* (Sheldrick, 2008 ▶); program(s) used to refine structure: *SHELXL97* (Sheldrick, 2008 ▶); molecular graphics: *PLATON* (Spek, 2009 ▶); software used to prepare material for publication: *SHELXL97*.

## Supplementary Material

Crystal structure: contains datablock(s) I, global. DOI: 10.1107/S1600536811029436/bt5581sup1.cif

Structure factors: contains datablock(s) I. DOI: 10.1107/S1600536811029436/bt5581Isup2.hkl

Supplementary material file. DOI: 10.1107/S1600536811029436/bt5581Isup3.cml

Additional supplementary materials:  crystallographic information; 3D view; checkCIF report

## Figures and Tables

**Table 1:** Hydrogen-bond geometry (Å, °)

*D*—H⋯*A*	*D*—H	H⋯*A*	*D*⋯*A*	*D*—H⋯*A*
N1—H1*N*⋯O2^i^	0.84 (2)	2.04 (2)	2.884 (2)	175 (2)
O3—H3*O*⋯O1	0.82 (2)	1.69 (2)	2.498 (2)	168 (3)

Symmetry code: (i) 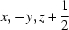.

**Fig. 1.**
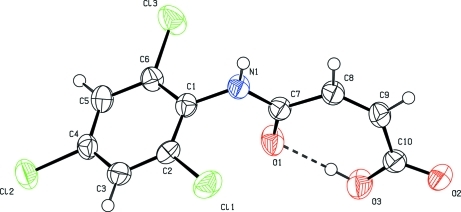
Molecular structure of (I), showing the atom labelling scheme and displacement ellipsoids are drawn at the 50% probability level.

**Fig. 2.**
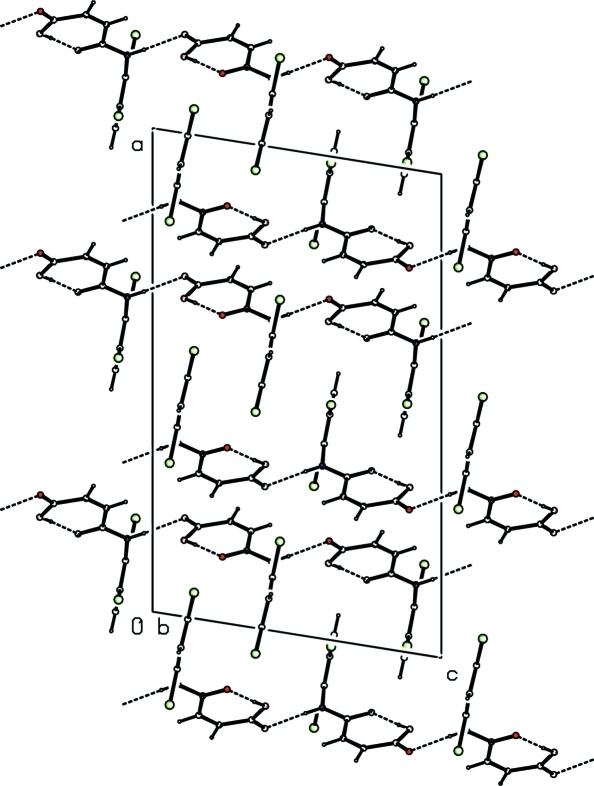
Molecular packing of (I) with hydrogen bonding shown as dashed lines.

## supplementary crystallographic information

### Comment

The amide moiety is an important constituent of many biologically significant compounds. As part of our studies on the effects of ring and side chain substitutions on the structures and other aspects of *N*-(aryl)-amides (Arjunan *et al.*, 2004; Bhat & Gowda, 2000; Gowda *et al.*, 2000, 2009; Prasad *et al.*, 2002) and *N*-(aryl)-methanesulfonamides (Jayalakshmi & Gowda, 2004), the crystal structure of *N*-(2,4,6-trimethylphenyl)-maleamic acid (I) has been determined. The conformation of the amide O atom is *anti* to the H atom attached to the adjacent C atom, while the carboxyl O atom is *syn* to the H atom attached to its adjacent C atom (Fig.1). The rare anti conformation of the C=O and O–H bonds of the acid group has been observed, similar to that obsrved in *N*-(2,4,6-trimethylphenyl)-maleamic acid (Gowda *et al.*, 2009) and *N*-phenylmaleamic acid (Lo & Ng, 2009), but contrary to the more general *syn* conformation observed for C=O and O–H bonds. The various modes of interlinking carboxylic acids by hydrogen bonds is described elsewhere (Leiserowitz, 1976).

The maleamic moiety includes a short intramolecular hydrogen O–H···O bond (Table 1). The C8–C9 bond length of 1.331 (3)Å clearly indicates the double bond character. The dihedral angle between the phenyl ring and the amido group –NHCO– is 83.2 (2)°. In the crystal structure, the intermolecular N–H···O hydrogen bonds link the molecules into column like chains along *b*-axis (Fig. 2).

### Experimental

The solution of maleic anhydride (0.025 mol) in toluene (25 ml) was treated dropwise with the solution of 2,4,6-trichloroaniline (0.025 mol) also in toluene (20 ml) with constant stirring. The resulting mixture was stirred for about 30 min and set aside for an additional 30 min at room temperature for the completion of reaction. The mixture was then treated with dilute hydrochloric acid to remove the unreacted 2,4,6-trichloroaniline. The resultant solid *N*-(2,4,6-trichlorophenyl)maleamic acid was filtered under suction and washed thoroughly with water to remove the unreacted maleic anhydride and maleic acid. It was recrystallized to constant melting point from ethanol. The purity of the compound was checked and characterized by its infrared spectra.

Prism like colorless single crystals used in X-ray diffraction studies were grown in an ethanol solution by slow evaporation at room temperature.

### Refinement

The H atoms of the NH group and the OH group were located in a difference map and later restrained to the distance N—H = 0.86 (2) Å and O—H = 0.82 (2) Å, respectively. The other H atoms were positioned with idealized geometry using a riding model with C—H = 0.93 Å. All H atoms were refined with isotropic displacement parameters set to 1.2 times of the *U*_eq_ of the parent atom.

### Crystal data

**Table:**

C_10_H_6_Cl_3_NO_3_	*F*(000) = 1184
*M**_r_* = 294.51	*D*_x_ = 1.650 Mg m^−^^3^
Monoclinic, *C*2/*c*	Mo *K*α radiation, λ = 0.71073 Å
Hall symbol: -C 2yc	Cell parameters from 2201 reflections
*a* = 21.928 (3) Å	θ = 2.6–27.9°
*b* = 8.2678 (8) Å	µ = 0.77 mm^−^^1^
*c* = 13.248 (2) Å	*T* = 293 K
β = 99.08 (1)°	Prism, colourless
*V* = 2371.7 (5) Å^3^	0.44 × 0.44 × 0.40 mm
*Z* = 8	

### Data collection

**Table:**

Oxford Diffraction Xcalibur diffractometer with a Sapphire CCD detector	2436 independent reflections
Radiation source: fine-focus sealed tube	2000 reflections with *I* > 2σ(*I*)
graphite	*R*_int_ = 0.012
Rotation method data acquisition using ω and φ scans	θ_max_ = 26.3°, θ_min_ = 2.6°
Absorption correction: multi-scan (*CrysAlis RED*; Oxford Diffraction, 2009)	*h* = −21→27
*T*_min_ = 0.729, *T*_max_ = 0.749	*k* = −9→10
4862 measured reflections	*l* = −14→16

### Refinement

**Table:**

Refinement on *F*^2^	Secondary atom site location: difference Fourier map
Least-squares matrix: full	Hydrogen site location: inferred from neighbouring sites
*R*[*F*^2^ > 2σ(*F*^2^)] = 0.035	H atoms treated by a mixture of independent and constrained refinement
*wR*(*F*^2^) = 0.094	*w* = 1/[σ^2^(*F*_o_^2^) + (0.0453*P*)^2^ + 2.1238*P*] where *P* = (*F*_o_^2^ + 2*F*_c_^2^)/3
*S* = 1.08	(Δ/σ)_max_ = 0.008
2436 reflections	Δρ_max_ = 0.55 e Å^−^^3^
161 parameters	Δρ_min_ = −0.44 e Å^−^^3^
2 restraints	Extinction correction: *SHELXL97* (Sheldrick, 2008), Fc^*^=kFc[1+0.001xFc^2^λ^3^/sin(2θ)]^-1/4^
Primary atom site location: structure-invariant direct methods	Extinction coefficient: 0.0069 (5)

### Special details

**Table:**

Geometry. All e.s.d.'s (except the e.s.d. in the dihedral angle between two l.s. planes) are estimated using the full covariance matrix. The cell e.s.d.'s are taken into account individually in the estimation of e.s.d.'s in distances, angles and torsion angles; correlations between e.s.d.'s in cell parameters are only used when they are defined by crystal symmetry. An approximate (isotropic) treatment of cell e.s.d.'s is used for estimating e.s.d.'s involving l.s. planes.
Refinement. Refinement of *F*^2^ against ALL reflections. The weighted *R*-factor *wR* and goodness of fit *S* are based on *F*^2^, conventional *R*-factors *R* are based on *F*, with *F* set to zero for negative *F*^2^. The threshold expression of *F*^2^ > σ(*F*^2^) is used only for calculating *R*-factors(gt) *etc*. and is not relevant to the choice of reflections for refinement. *R*-factors based on *F*^2^ are statistically about twice as large as those based on *F*, and *R*- factors based on ALL data will be even larger.

### Fractional atomic coordinates and isotropic or equivalent isotropic displacement parameters (Å^2^)

**Table:**

	*x*	*y*	*z*	*U*_iso_*/*U*_eq_	
Cl1	0.01367 (3)	0.14374 (7)	0.38180 (5)	0.0547 (2)	
Cl2	−0.05298 (3)	0.76688 (8)	0.35643 (5)	0.0546 (2)	
Cl3	0.18696 (3)	0.61105 (7)	0.44095 (6)	0.0594 (2)	
O1	0.13622 (8)	0.26049 (19)	0.24188 (11)	0.0489 (4)	
O2	0.20438 (7)	−0.1698 (2)	0.11143 (12)	0.0491 (4)	
O3	0.15810 (9)	0.0634 (2)	0.10918 (12)	0.0554 (5)	
H3O	0.1526 (15)	0.138 (3)	0.147 (2)	0.083*	
N1	0.14268 (8)	0.2673 (2)	0.41225 (12)	0.0356 (4)	
H1N	0.1593 (10)	0.234 (3)	0.4704 (14)	0.043*	
C1	0.09629 (9)	0.3880 (2)	0.40607 (13)	0.0325 (4)	
C2	0.03409 (10)	0.3452 (2)	0.38660 (14)	0.0355 (4)	
C3	−0.01220 (9)	0.4607 (3)	0.37139 (15)	0.0389 (5)	
H3	−0.0535	0.4305	0.3569	0.047*	
C4	0.00448 (9)	0.6214 (3)	0.37825 (14)	0.0362 (5)	
C5	0.06509 (10)	0.6697 (3)	0.40216 (16)	0.0394 (5)	
H5	0.0753	0.7787	0.4096	0.047*	
C6	0.11040 (9)	0.5516 (2)	0.41482 (15)	0.0358 (4)	
C7	0.15744 (9)	0.2035 (2)	0.32652 (15)	0.0344 (4)	
C8	0.19895 (10)	0.0623 (3)	0.34006 (15)	0.0391 (5)	
H8	0.2183	0.0426	0.4066	0.047*	
C9	0.21238 (10)	−0.0406 (3)	0.26948 (16)	0.0407 (5)	
H9	0.2405	−0.1206	0.2950	0.049*	
C10	0.19092 (9)	−0.0518 (3)	0.15725 (15)	0.0371 (5)	

### Atomic displacement parameters (Å^2^)

**Table:**

	*U*^11^	*U*^22^	*U*^33^	*U*^12^	*U*^13^	*U*^23^
Cl1	0.0623 (4)	0.0373 (3)	0.0631 (4)	−0.0113 (3)	0.0060 (3)	−0.0095 (3)
Cl2	0.0499 (3)	0.0574 (4)	0.0557 (4)	0.0228 (3)	0.0064 (3)	−0.0018 (3)
Cl3	0.0376 (3)	0.0433 (3)	0.0960 (5)	−0.0025 (2)	0.0072 (3)	0.0033 (3)
O1	0.0625 (10)	0.0509 (10)	0.0327 (8)	0.0257 (8)	0.0056 (7)	0.0070 (7)
O2	0.0507 (9)	0.0483 (9)	0.0485 (9)	0.0001 (7)	0.0086 (7)	−0.0150 (7)
O3	0.0717 (12)	0.0589 (11)	0.0340 (8)	0.0200 (9)	0.0034 (7)	−0.0018 (7)
N1	0.0442 (10)	0.0324 (9)	0.0292 (8)	0.0089 (7)	0.0026 (7)	0.0037 (7)
C1	0.0398 (10)	0.0322 (10)	0.0263 (9)	0.0039 (8)	0.0073 (7)	0.0011 (8)
C2	0.0437 (11)	0.0346 (10)	0.0288 (9)	−0.0037 (8)	0.0075 (8)	−0.0044 (8)
C3	0.0345 (10)	0.0498 (13)	0.0330 (10)	−0.0009 (9)	0.0073 (8)	−0.0067 (9)
C4	0.0391 (11)	0.0403 (11)	0.0302 (10)	0.0108 (9)	0.0085 (8)	−0.0009 (8)
C5	0.0453 (12)	0.0307 (10)	0.0433 (11)	0.0040 (9)	0.0108 (9)	0.0018 (9)
C6	0.0351 (10)	0.0341 (10)	0.0390 (11)	0.0007 (8)	0.0082 (8)	0.0021 (8)
C7	0.0379 (10)	0.0326 (10)	0.0322 (10)	0.0033 (8)	0.0039 (8)	0.0019 (8)
C8	0.0456 (12)	0.0380 (11)	0.0315 (10)	0.0102 (9)	−0.0006 (8)	0.0018 (8)
C9	0.0425 (12)	0.0352 (11)	0.0429 (11)	0.0107 (9)	0.0018 (9)	0.0007 (9)
C10	0.0320 (10)	0.0415 (11)	0.0387 (11)	−0.0035 (9)	0.0087 (8)	−0.0040 (9)

### Geometric parameters (Å, °)

**Table:**

Cl1—C2	1.723 (2)	C2—C3	1.385 (3)
Cl2—C4	1.733 (2)	C3—C4	1.377 (3)
Cl3—C6	1.731 (2)	C3—H3	0.9300
O1—C7	1.237 (2)	C4—C5	1.376 (3)
O2—C10	1.210 (3)	C5—C6	1.384 (3)
O3—C10	1.299 (3)	C5—H5	0.9300
O3—H3O	0.819 (18)	C7—C8	1.473 (3)
N1—C7	1.338 (3)	C8—C9	1.331 (3)
N1—C1	1.418 (2)	C8—H8	0.9300
N1—H1N	0.843 (16)	C9—C10	1.490 (3)
C1—C6	1.388 (3)	C9—H9	0.9300
C1—C2	1.393 (3)		
			
C10—O3—H3O	112 (2)	C4—C5—H5	120.9
C7—N1—C1	119.74 (16)	C6—C5—H5	120.9
C7—N1—H1N	121.4 (16)	C5—C6—C1	122.08 (19)
C1—N1—H1N	118.8 (16)	C5—C6—Cl3	118.60 (16)
C6—C1—C2	117.51 (18)	C1—C6—Cl3	119.32 (15)
C6—C1—N1	122.16 (18)	O1—C7—N1	120.82 (18)
C2—C1—N1	120.28 (18)	O1—C7—C8	123.28 (18)
C3—C2—C1	121.69 (19)	N1—C7—C8	115.89 (17)
C3—C2—Cl1	118.74 (16)	C9—C8—C7	128.44 (19)
C1—C2—Cl1	119.58 (16)	C9—C8—H8	115.8
C4—C3—C2	118.33 (19)	C7—C8—H8	115.8
C4—C3—H3	120.8	C8—C9—C10	132.11 (19)
C2—C3—H3	120.8	C8—C9—H9	113.9
C5—C4—C3	122.17 (19)	C10—C9—H9	113.9
C5—C4—Cl2	119.15 (16)	O2—C10—O3	120.37 (19)
C3—C4—Cl2	118.69 (16)	O2—C10—C9	119.2 (2)
C4—C5—C6	118.12 (19)	O3—C10—C9	120.47 (18)
			
C7—N1—C1—C6	−98.7 (2)	C4—C5—C6—Cl3	178.00 (15)
C7—N1—C1—C2	78.7 (2)	C2—C1—C6—C5	−1.7 (3)
C6—C1—C2—C3	3.1 (3)	N1—C1—C6—C5	175.71 (18)
N1—C1—C2—C3	−174.36 (17)	C2—C1—C6—Cl3	179.01 (14)
C6—C1—C2—Cl1	−176.52 (14)	N1—C1—C6—Cl3	−3.5 (3)
N1—C1—C2—Cl1	6.0 (2)	C1—N1—C7—O1	8.1 (3)
C1—C2—C3—C4	−1.5 (3)	C1—N1—C7—C8	−170.97 (18)
Cl1—C2—C3—C4	178.18 (15)	O1—C7—C8—C9	−11.8 (4)
C2—C3—C4—C5	−1.7 (3)	N1—C7—C8—C9	167.2 (2)
C2—C3—C4—Cl2	178.53 (14)	C7—C8—C9—C10	−1.0 (4)
C3—C4—C5—C6	3.0 (3)	C8—C9—C10—O2	−170.9 (2)
Cl2—C4—C5—C6	−177.20 (15)	C8—C9—C10—O3	9.0 (4)
C4—C5—C6—C1	−1.2 (3)		

### Hydrogen-bond geometry (Å, °)

**Table:**

*D*—H···*A*	*D*—H	H···*A*	*D*···*A*	*D*—H···*A*
N1—H1N···O2^i^	0.84 (2)	2.04 (2)	2.884 (2)	175 (2)
O3—H3O···O1	0.82 (2)	1.69 (2)	2.498 (2)	168 (3)

Symmetry codes: (i) *x*, −*y*, *z*+1/2.
